# Knockdown of a β-Adrenergic-Like Octopamine Receptor Affects Locomotion and Reproduction of *Tribolium castaneum*

**DOI:** 10.3390/ijms22147252

**Published:** 2021-07-06

**Authors:** Li-Sha Zheng, Xiao-Qiang Liu, Ge-Ge Liu, Qian-Qiao Huang, Jin-Jun Wang, Hong-Bo Jiang

**Affiliations:** 1 Key Laboratory of Entomology and Pest Control Engineering, College of Plant Protection, Southwest University, Chongqing 400715, China; zls1582@email.swu.edu.cn (L.-S.Z.); xqlcoin2018@email.swu.edu.cn (X.-Q.L.); lgg2017@email.swu.edu.cn (G.-G.L.); rachel980105@email.swu.edu.cn (Q.-Q.H.); wangjinjun@swu.edu.cn (J.-J.W.); 2 Academy of Agricultural Sciences, Southwest University, Chongqing 400715, China; 3 Laboratory of Integrated Pest Management on Tropical Crops, Environment and Plant Protection Institute, Chinese Academy of Tropical Agriculture Sciences, Ministry of Agriculture, Haikou 570100, China

**Keywords:** red flour beetle, octopamine receptor, locomotion, reproduction

## Abstract

The neurohormone octopamine regulates many crucial physiological processes in insects and exerts its activity via typical G-protein coupled receptors. The roles of octopamine receptors in regulating behavior and physiology in Coleoptera (beetles) need better understanding. We used the red flour beetle, *Tribolium castaneum*, as a model species to study the contribution of the octopamine receptor to behavior and physiology. We cloned the cDNA of a β-adrenergic-like octopamine receptor (*TcOctβ2R*). This was heterologously expressed in human embryonic kidney (HEK) 293 cells and was demonstrated to be functional using an in vitro cyclic AMP assay. In an RNAi assay, injection of dsRNA demonstrated that *TcOctβ2R* modulates beetle locomotion, mating duration, and fertility. These data present some roles of the octopaminergic signaling system in *T. castaneum*. Our findings will also help to elucidate the potential functions of individual octopamine receptors in other insects.

## 1. Introduction

Octopamine (OA) was first discovered in the salivary glands of an octopus [1], and it shares structural and functional similarities to the vertebrate biogenic amines, adrenaline, and noradrenaline. High concentrations of OA have been found in the neuronal and nonneuronal tissues of most invertebrate species [2]. OA acts as a neurohormone, neuromodulator, and neurotransmitter in invertebrates [3], with essential functions and regulation of many physiological processes, including olfactory sensitivity [4,5,6], endocrine regulation [7], learning and memory [8,9], locomotion [10,11,12,13], sleep [14], induction of germline stem cell increase [15], and ovulation [16,17,18,19].

OA acts by binding to typical G-protein coupled receptors (GPCRs) with seven conserved transmembrane domains. The first insect OA receptor was isolated from *Drosophila melanogaster* [20]. Subsequently, a number of OA receptors were cloned from other insect species. According to their functional similarities to vertebrate adrenergic receptors, in terms of amino acid sequence and signaling pathway, OA receptors are classified into four major groups designated as α_1_-adrenergic-like OA receptors (Octα_1_-R), α_2_-adrenergic-like OA receptors (Octα_2_-R), β-adrenergic-like OA receptors (Octβ1-R, Octβ2-R, Octβ3-R), and OA/tyramine receptors (Tyr1-R, Tyr2-R, Tyr3-R) [21,22,23]. Activation of Octα_1_-R expressed in cell lines primarily leads to an increase in both intracellular Ca^2+^ and cAMP concentration, while activation of Octα_2_-R leads to an increase in intracellular Ca^2+^ and the decrease of cAMP concentration. Activation of Octβ-Rs only induces an increase in intracellular cAMP concentration [21]. The Tyr1-R and Tyr3-R class of receptors can be stimulated by both tyramine and OA. The activation of these receptors results in the inhibition of the cAMP level and an increase in intracellular Ca^2+^ concentration. The difference between these two types of TyrRs lies in their affinities for OA and TA when the intracellular Ca^2+^ concentration changes. The Tyr2-Rs have been characterized in *Drosophila* and *Bombyx mori*. They are specifically activated by tyramine and selectively coupled to intracellular Ca^2+^ mobilization but have no effect on intracellular cAMP concentration [21,23,24].

The functions of OA receptors, especially OctβRs, have been studied in many insect species. Octβ2R is expressed in the female reproductive tract controlling ovulation and fertilization in *D. melanogaster* [16,19]. Studies on *Nilaparvata lugens* have shown that the injection of OAR antagonists, and blockage of *N1OA2B2* both lead to a decrease in egg production [18]. The octopamine receptor antagonists (mianserin and phentolamine) impaired the movement of adult rice stem borers, *Chilo suppressalis*, probably due to the inhibition of CsOA2B2 [25]. The activation of distinct OA receptors in skeletal and cardiac muscles is necessary for *Drosophila* exercise adaptations, and the expression of Octβ2R in skeletal muscles is required for improving endurance and speed [13]. As a potential insecticide target, OctβRs have been studied for their pharmacological characterizations. The interaction of OctβRs on agonists and antagonists was tested in *Plutella xylostella* [26], *B. mori* [27], *N. lugens* [18], *Rhodnius prolixus* [24], and *Nephotettix cincticeps* [28].

It has been well documented that octopamine receptors have diverse functions in different insects. The physiological functions of the octopamine receptors have been well characterized in *Drosophila* model systems [16]. However, there is no functional study on the octopamine receptors in coleopteran insects yet. The phylogenetic comparison, tissue expression profiles, and flexible behavior-related gene expression of OA receptors were studied in the subsocial burying beetle, *Nicrophorus vespilloides* [29,30]. With the availability of genome information and the high efficiency of RNA interference (RNAi), the red flour beetle, *Tribolium castaneum,* is a good model system for functional genomic studies. Furthermore, it has been reported that the duration of tonic immobility was shortened in a dose-dependent manner by injection of OA [31], showing that OA is involved in *T. castaneum* behavior. Therefore, we combined physical cloning, transcriptional expression profiling, heterologous expression, and RNAi assays to characterize the functionality of *TcOctβ2R* in *T. castaneum*. We sought to determine the contribution of the OA receptor to beetle behavior and physiology.

## 2. Results

### 2.1. Molecular Cloning and Sequence Analysis

**Figure 1:**
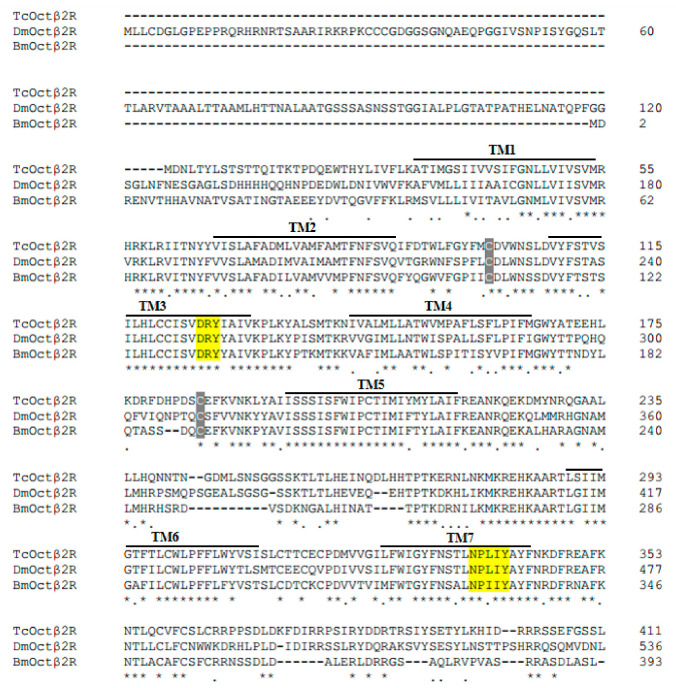
Multiple sequence alignment of TcOctβ2R with two β-adrenergic-like receptors from *D. melanogaster* (DmOctβ2R) and *B. mori* (BmOctβ2R). TM means transmembrane domains. The seven transmembrane domains are numbered as TM1–7. Identical amino acids are marked by asterisks, and conserved amino acids are marked by dots. The deep grey background indicates conserved cysteine residues. The yellow background indicates a conserved amino acid motif.

The open reading frame (ORF) of *TcOctβ2R* (GenBank Accession Number: NM_001293572) is 1236 bp and encodes a protein of 411 amino acids with a molecular weight of 47.9 kD and an isoelectric point of 8.71. The prediction of the transmembrane structure shows that TcOctβ2R is a GPCR with typical seven transmembrane domains. The comparison of the amino acid sequence of TcOctβ2R with DmOctβ2R and BmOctβ2R showed a similarity of 51.5%. Through multiple sequence alignment, it is found that the cysteine residues indicated by the deep grey background are highly conserved in the extracellular II and III of Octβ2R. In addition, TcOctβ2R has the conserved DRY motif in transmembrane domain III (TM3) and NPxxY motif in TM7. These two motifs are necessary for G protein coupling and are conserved in all adrenergic receptors (Figure 1).

**Figure 2:**
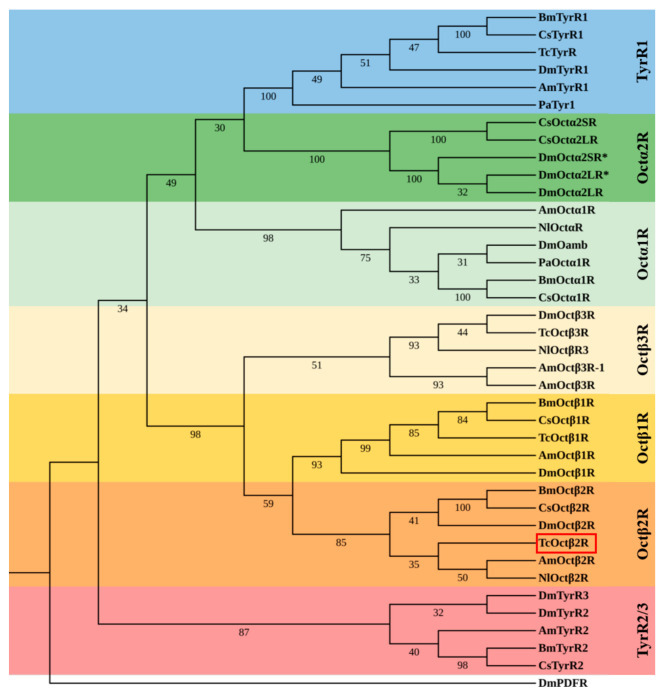
Phylogenetic tree of TcOctβ2R (marked by the red square) and various biogenic amine receptors. The neighbor-joining tree was constructed in MEGA 5.0 using 1000 bootstrap tests. The numbers at the nodes of the branches represent the level of bootstrap support for each branch. The *Drosophila* PDF receptor served as the out-group. Am, *A. mellifera*; Bm, *B. mori*; Dm, *D. melanogaster*; Cs, *C. suppressalis*; Nl, *N. lugens*; Pa, *Periplaneta americana*; Tc, *T. castaneum*.The accession numbers of all receptors used in the phylogenetic analysis can be found in Table S2.

A phylogenetic tree constructed with the insect octopamine receptors clustered into three groups, consisting of octopamine/tyramine receptors, OctαRs, and OctβRs (Figure 2). TcOctβ2R was grouped with Octβ2Rs and showed a close relationship to AmOctβ2R of *Apis mellifera* and NIOctβ2R of *N. lugens* (Figure 2).

### 2.2. Heterologous Expression and Functional Assay

**Figure 3:**
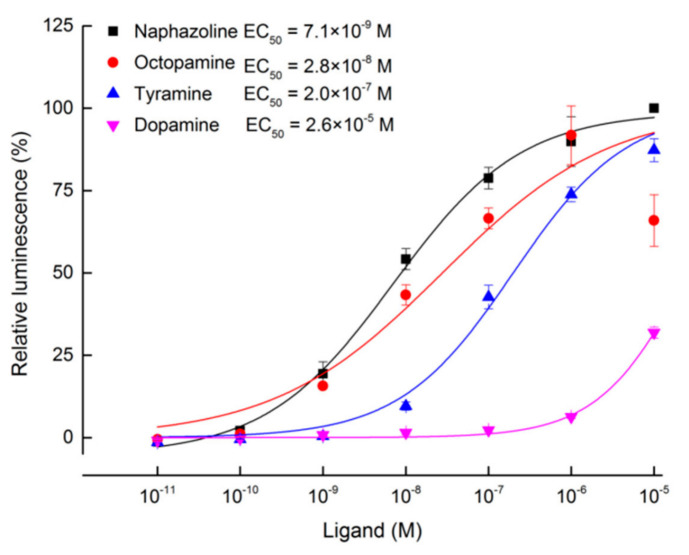
Dose–response curves of TcOctβ2R transiently expressed cells (HEK 293) to four tested ligands (naphazoline, octopamine, tyramine, and dopamine). Each spot represents the mean relative luminescence ± S.E. from three biological replications. The relative luminescence was normalized to the luminescence caused by the application of naphazoline at 1.0 × 10^−5^ M.

Transfection of pcDNA3.1(+)-*TcOctβ2R* plasmid makes human embryonic kidney (HEK 293) cells transiently express TcOctβ2R on the cell membrane. The activation of the TcOctβ2R on the membrane by the ligand will cause the accumulation of cAMP, which manifests as an increased luminescence of the GloSensor. We examined the activity of different biogenic amines, including naphazoline, OA, TA, and dopamine, on the receptor. In the assays, no stimulation of cAMP production was recorded after incubation with 1.0 × 10^−6^ M biogenic amines on an empty pcDNA3.1(+) vector. In contrast, naphazoline, OA, and TA significantly induced the increase of cAMP in cells expressing TcOctβ2R (Figure 3). Among the tested chemicals, naphazoline showed the most potent activation on TcOctβ2R with a very low effective concentration (EC_50_) of 7.1 × 10^−9^ M. The model ligand OA also showed a very potent activation with an EC_50_ of 2.8 × 10^−8^ M. TA showed a moderate activation with an EC_50_ of 2.0 × 10^−7^ M. However, dopamine did not activate TcOctβ2R at low concentrations, and activation only occurred at the highest concentration (1.0 × 10^−5^ M) tested.

### 2.3. Spatial and Temporal Expression Profiles

**Figure 4:**
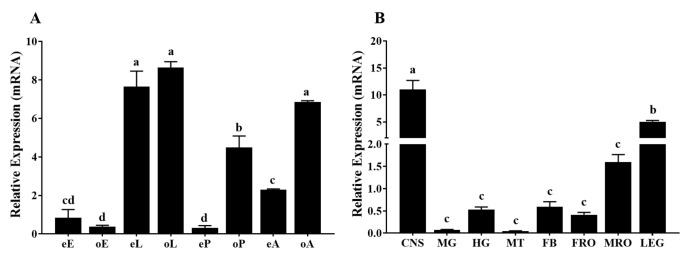
(**A**) Relative expression levels of *TcOctβ2R* at different developmental stages. Different stages are denoted by capitalized letters: E (egg), L (larva), P (pupa), and A (adult). Letters in lowercase e and o represent early and old, respectively; (**B**) relative expression levels of *TcOctβ2R* in various tissues of adults. CNS, central nervous system; FB, fat body; MG, midgut; MT, Malpighian tubules; FRO, female reproductive organs; MRO, male reproductive organs; LEG, legs. All the data shown are means of the relative expression ± standard error (S.E.) (*n* = 4), normalized to *RPS3* transcript levels. Different letters on the bar represent a significant difference in ANOVA (Tukey, *p* < 0.05).

Based on the standard curves obtained by the serial dilutions of cDNA, the primer efficiencies were 95.2% and 98.6% for *RPS3* and *TcOctβ2R*, respectively. The RT-qPCR results showed that transcripts of *TcOctβ2R* were detected across all developmental stages tested (Figure 4A). The highest expression was observed in the larval stage and old adult stage, followed by the old pupal and early adult stages. The lowest expression occurred in the early pupal and egg stages. Except for the egg stage, the expression level of *TcOctβ2R* at other developmental stages showed a tendency for having higher expression in the old stage than in the early stage. Among the different tissues of the 7-d-old virgin adults, a significantly higher expression level of *TcOctβ2R* was recorded in the central nervous system (CNS, including the brain, thoracic, and abdominal ganglia) (Figure 4B). No significant difference was found among the expression levels of *TcOctβ2R* in the other tissues.

### 2.4. Effect of TcOctβ2R Knockdown on Mobility

**Figure 5:**
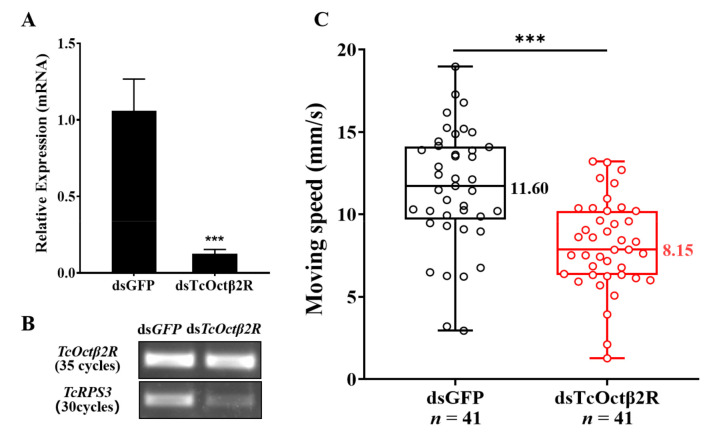
RNAi of *TcOctβ2R* and mobility assay: (**A**) RNAi efficiency tested by qRT-PCR; (**B**) the confirmation of RNAi efficiency by RT-PCR; (**C**) moving speed (in millimeters per second) of *T. castaneum*. Asterisks represent the significant difference in independent *t*-test (*n* = 41, *** *p* < 0.001).

Injection of dsRNA into pupae targeting *TcOctβ2R* significantly suppressed its expression in adults (Figure 5A). The transcription of *TcOctβ2R* was significantly reduced by 88.2%. The reduction was confirmed by regular RT-PCR, where the dsRNA-treated group showed a very faded band on an agarose gel (Figure 5B). In the adult mobility assay, 82 individuals, divided into two groups (control insects and ds *TcOctβ2R*-treated), were tested. The moving speed of each beetle was recorded in millimeters per second. As shown in Figure 5C, the average speed of 41 individuals from the ds*TcOctβ2R*-injected group was significantly decreased by nearly 30%, compared to the control group (*p* < 0.01, independent *t*-test).

### 2.5. Effect of TcOctβ2R Knockdown on Mating Behavior and Reproduction

**Figure 6:**
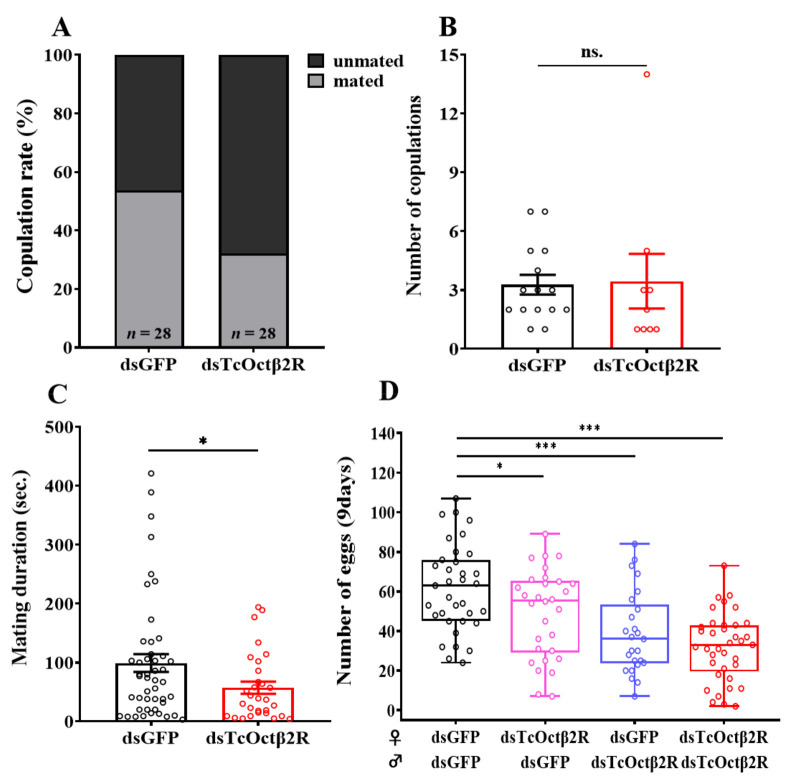
Mating behavior and fecundity assay after RNAi: (**A**) copulation rate (%); (**B**) number of copulations (mean ± S.E.) in 90 min; (**C**) mating duration (*n* = 28); (**D**) total number of eggs laid in 9 d (*n* = 13–14, mean ± S.E.). Asterisks represent the significant difference in independent *t*-test (*n* = 25–37, * *p* < 0.05, *** *p*<0.001).

By observing the mating behavior of beetles, it was found that the copulation rate of the ds*TcOctβ2R*-injected group was 32.1% less than the 53.6% of the ds*GFP*-injected group (Figure 6A). For mated beetles, *TcOctβ2R* knockdown did not affect the number of copulations (Figure 6B). The ds*TcOctβ2R*-injected group, however, had significantly reduced mating duration, compared to the control group (Figure 6C). The average mating duration of the ds*TcOctβ2R*-injected beetles was 54.8 ± 10.4 s, which was about 44.2 s less than that of the ds*GFP*-injected beetles.

As *TcOctβ2R* knockdown inhibits beetle mating behavior, cross-mating experiments after RNAi were used to test the effect on fecundity. Based on the total number of eggs laid in 9 d, the rankings were in the following order (high to low): *dsGFP*♀x*dsGFP*♂> ds*TcOctβ2R*♀x*dsGFP*♂> *dsGFP*♀xds*TcOctβ2R*♂> ds*TcOctβ2R*♀xds*TcOctβ2R*♂(Figure 6D). Combinations of single-pair mating in which either sex was treated with *dsTcOctβ2R* also showed a significant reduction in the total eggs laid (27–57% of the control), regardless of which sex was treated. Therefore, the silencing of *TcOctβ2R* affects both the mating behavior and fertility of beetles. In addition, the silencing of *TcOctβ2R* had a greater impact on male fertility than on female fertility, which coincides with the higher expression of this gene in the testis.

## 3. Discussion

The availability of well-annotated genome information of model organisms, such as *T. castaneum*, provided an opportunity to study the functions of the octopaminergic signaling system. Octopamine receptors have been identified from many species, including *B. mori* [2,32], *D. melanogaster* [33], *P. americana* [34], *A. mellifera* [35], *Bactrocera dorsalis* [36], *P. xylostella* [26], and *R. prolixus* [24]. In the current study, we cloned an octopamine receptor from *T. castaneum*, and the phylogenetic analysis indicated that it belongs to the family of insect OctβRs, which are structurally similar to the vertebrate β-adrenergic receptors.

Studies on the agonist or antagonist profiles of octopamine receptors have suggested their potential as targets for novel insecticides [22]. The in vitro agonist assays of octopamine receptors have been successfully performed in *D. melanogaster* [37] and *B. dorsalis* [36]. In our study, the rank order for the potency of the tested ligands was as follows: naphazoline > OA > TA > dopamine. Naphazoline also has significant agonistic effects on BdOctβR1 [36]. OA possessed the highest agonistic activity against DmOctβ2R [37]. The EC_50_ value of OA in *T. castaneum* was 2.8 × 10^−8^ M, and it was less potent than DmOctβ2R (EC_50_: 1.53 × 10^−8^ M) [37]. In *A. mellifera*, the EC_50_ of OA for AmOctβ2R was 1.82 × 10^−9^ M [35]. This suggests that AmOctβ2R or DmOctβ2R may have a better coupling with G-proteins than TcOctβ2R.

The transcriptional profiles of *TcOctβ2R* revealed ubiquitous expression in all developmental stages and tissues examined. *TcOctβ2R* was highly expressed in the larval and old adult stages (Figure 4A). Similarly, *DmOctβ2R*, *CsOctβ2R*, and *MsOA2B2* exhibited high expression in larvae [25,37,38]. *PxOA2B2* and *NiOA2B2* were highly expressed in male adults but not in female adults. However, current research on Octβ2Rs is focused on effects on female fecundity, while male-specific behaviors have not been studied well. *TcOctβ2R* was highly expressed in the *T. castaneum* CNS. Similar results were found in *DmOctβ2R* [37] and *CsOctβ2R* [25], which are highly expressed in heads. In addition, the distribution pattern of *NvOctβ2R* indicated that it is expressed predominantly in the thoracic musculature [29]. The highest transcript levels of *SgOctβR* were found in the flight muscles, followed by the CNS, which were determined to be associated with flight ability [39]. Here, we found that *TcOctβ2R* was highly expressed in the CNS, followed by the legs and male reproductive organs (Figure 4B). Therefore, we conclude that *TcOctβ2R* mainly acts as a neurotransmitter receptor in the nervous system of *T. castaneum*. It may also be involved in behaviors regulated by the peripheral nervous system.

In *D. melanogaster*, OA neurons regulate the expansion of excitatory glutamatergic neuromuscular arbors through DmOctβ2R on glutamatergic motor neurons [40]. This indicated that this subtype of OA receptors might be important in locomotion. *Octβ2R* is strongly expressed in the skeletal muscle system of *Drosophila* larvae. In adults, *Octβ2R* is highly expressed in the leg skeletal muscles and longitudinal muscles in the abdomen. An octopaminergic system is involved in the regulation of prothoracicotropic hormone (PTTH) and insulin-like peptides (ILPs) signaling [41], which further regulate the energy metabolism in insects [42]. In the current study, we found that knockdown of *TcOctβ2R* reduced the locomotory activity in *T. castaneum*. This could be achieved by participating in the control of skeletal muscle contraction and the hormonal regulation of energy metabolism through various neuronal signaling systems.

Octβ2R plays an important role in female reproductive behavior. OA regulates the contraction of muscles in female reproductive organs through the abdominal ganglia (Abg) octopaminergic neurons. These muscles occupy specific locations in the reproductive system and affect the release of sperm from spermathecae and ovulation [43]. In *Drosophila*, the combination of OA with Octb2R and OAMB in epithelial cells induces the transport of eggs from the ovary to the uterus. Activation of OAMB induces an increase in cytoplasmic Ca^2+^ levels and stimulates the production of secretions required for ovulation. Octb2R activation induces oviduct muscle relaxation by increasing cAMP levels [16]. Similarly, *NIOAB2B* is involved in regulating ovulation in *N. lugens*. The RNAi of *NIOAB2B* can cause ovaries to increase in size due to egg retention [18]. In the present study on *T. castaneum*, the total number of eggs laid by ds*TcOctβ2R*-injected females in 9 d was significantly lower than the number of eggs laid by control beetles (Figure 6D). In addition, *TcOctβ2R* RNAi has a greater impact on male reproduction than female (Figure 6D). In general, there is a positive correlation between mating duration and semen delivery [44]. Since *TcOctβ2R* RNAi shortens the mating duration of males, this could decrease the amount of sperm delivered to females and lead to a decrease in egg production. *Octβ2R* is also highly expressed in the male reproductive organs of *T. castaneum* (Figure 4B) and *N. lugens* [45]. Knockdown of *TcOctß2R* hinders the OA signal in the male reproductive organs, and this may reduce the transmission of semen by affecting the contraction of the ejaculatory duct muscles, which reduces the number of eggs produced by females. Thus, it is necessary to further study how *TcOctß2R* affects the process of male ejaculation.

In summary, the cDNA of a β-adrenergic-like octopamine receptor (*TcOctβ2R*) was cloned from *T. castaneum*. It was functionally identified by heterologous expression and an in vitro cyclic AMP assay. In RNAi assays, dsRNA injection indicated that this receptor modulates beetle locomotion, mating behavior, and fertility. These findings will help to elucidate the functions of individual OA receptors in beetles and in other insects.

## 4. Materials and Methods

### 4.1. Test Insects

The Georgia-1 (GA1) strain of *T. casraneum* (obtained from Dr. Yoonseong Park of Kansas State University, Manhattan, KS, USA) was reared in wheat flour and brewer yeast powder (10:1) at 30 °C, a 16:8 h (L:D) photoperiod and 30% relative humidity.

### 4.2. Primers, Plasmids, and Chemicals

Primers (Table S1) used in this manuscript were designed based on the predicted sequence of *T. castaneum* and synthesized by Invitrogen (Shanghai, China). The pGEM-T Easy Vector (Promega, Madison, WI, USA) was used to clone the PCR amplicon of *TcOctβ2R*. The expression vector pcDNA3.1(+) was a gift from Dr. Yoonseong Park of Kansas State University. High-quality plasmid DNA prepared by a QIAGEN Plasmid Plus Midi Kit (Hilden, Germany) was used for transient transfection and heterologous expression.

The HEK 293 cells were cultured adherently in a culture medium at 37 °C with 5% CO_2_ content. The culture medium was composed of DMEM/F12 medium, 10% fetal bovine serum (FBS), 1% fungizone, and 1% penicillin/streptomycin. Coelenterazine h and the reagents used for cell culture were purchased from Gibco Life Technologies (Grand Island, NY, USA). The TransIT–LT1 transfection reagent used for the transient transfections was purchased from Mirus Bio Chemicals (Madison, WI, USA). OA hydrochloride, dopamine hydrochloride, TA hydrochloride, forskolin, and naphazoline hydrochloride were all purchased from Sigma-Aldrich (St. Louis, MO, USA). The GloSensor reagent used for the cAMP assay was purchased from Promega.

### 4.3. Molecular Cloning and Sequence Analysis

Total RNA was isolated from the whole body of *T. castaneum* adults using TRIZOL reagent according to the manufacturer protocol. The first-strand cDNA was synthesized by the PrimeScript first-strand synthesis system (TaKaRa, Dalian, China) after digesting genome DNA with RQ1 RNase-Free DNase (Promega). The ORF of *TcOctß2R* was amplified by a nested PCR using high fidelity DNA polymerase PrimeSTAR HS (Takara). The PCR conditions were as follows: 98 °C for 2 min, 35 cycles at 98 °C for 10 s, 60 °C for 15 s, and 72 °C for 90 s, and final extension at 72 °C for 10 min. The purified PCR product was cloned into the pGEM-T Easy vector (Promega) and sequenced.

Nucleotide sequence and putative protein sequence of the *Tribolium* Octβ2R receptor were analyzed using DNAMAN7 (Lynnon BioSoft, Vaudreuil, QC, Canada). The isoelectric point and molecular weight of the putative protein were predicted on the ExPASy Proteomics Server (http://cn.expasy.org/tools/pi_tool, access on 29 June 2021). Similar sequences were obtained by a BlastP search against the nonredundant protein database on NCBI (http://www.ncbi.nlm.nih.gov, access on 29 June 2021). Multiple alignments of the related sequences were conducted using ClustalW2 (http://www.ebi.ac.uk/Tools/msa/clustalw2, access on 29 June 2021). Transmembrane helices were predicted using the TMHMM server (http://www.cbs.dtu.dk/services/TMHMM, access on 29 June 2021). Phylogenetic analysis was performed with MEGA 5.0 using the neighbor-joining method and 1000 bootstrap tests. The pigment-dispersing factor receptor (PDF receptor) in *D. melanogaster* served as an out-group.

### 4.4. Heterologous Expression and Functional Assay

The *TcOctβ2R*-pGEMT was subcloned into the pcDNA3.1(+) vector by a NotI digestion and ligation. HEK 239 cells were used for the heterologous expression. Briefly, cells were transfected using the TransIT–LT1 transfection reagent purchased from Mirus Bio LLC. At 36 h after the transfection, the cells were collected. They were further preincubated with the GloSensor reagent (Promega) for an additional 2 h for the cyclic AMP (cAMP) assay, as described previously [46]. Tenfold serial dilutions of tested ligands, including OA, TA, dopamine, and naphazoline, were applied to the cells. Forskolin at 10 μM served as a positive control for the receptor activation. The test ligands diluted with DMEM/F12 (Gibco Life Technologies) were added to the wells of the 96-well plate, and then, 50 uL of cells were injected into the wells, and the luminescence was detected. The elevated luminescence levels caused by the cAMP accumulation were measured within 15 min in 30 s intervals using a TriStar^2^ LB 942 Multimode Reader (Berthold Technologies, Bad Wildbad, Germany). The luminescence for each tested ligand was normalized to the luminescence produced by naphazoline at the concentration of 10 μM, which was employed as the model ligand and set as 100% of response, after background subtractions. Based on the relative luminescence, logistic fitting in Origin 8.6 (OriginLab, Northampton, MA, USA) generated a dose–response curve of the receptor to each tested ligand. All experiments were conducted using three biological replicates.

### 4.5. Quantitative Reverse Transcription PCR (qRT-PCR)

Beetles at different developmental stages were collected, as described previously, for developmental expression profiling [47]. Different tissues were dissected from 7-d-old virgin adults: CNS (including the brain, thoracic, and abdominal ganglia), midgut, hindgut, Malpighian tubules, legs, male reproductive organs, and female reproductive organs. In total, 20 adults were pooled to prepare the midgut, hindgut, leg, and male and female reproductive organs, while 40 individuals were pooled to collect the Malpighian tubules and CNS. Total RNA extraction and cDNA synthesis were performed as described above. The qRT-PCR primers are listed in Table S1. A threefold serial dilution of the cDNA was used to obtain the standard curve for calculating the amplification efficiency of each primer pair. qRT-PCR was performed using the IQ™ SYBR^®^ Green Supermix (Promega) on a Stratagene Mx3000P system (Stratagene, La Jolla, CA, USA). The reference gene ribosomal protein S3 (rpS3, GenBank Accession Number CB335975) was used to calculate the relative expression of *TcOctβ2R* with qBase^+^ software [48]. All experiments had four biological replications.

### 4.6. RNA Interference

Primers (Table S1) tailing the T7 promoter were used to amplify the target region for the synthesis of gene-specific dsRNA. The dsRNA synthesis was conducted using a TranscriptAid T7 High Yield Transcription Kit (Thermo Fisher Scientific, Waltham, MA, USA). For RNAi, a total of 200 ng dsRNA was injected into the beetle body cavity. Early pupae (within 24 h after pupation) were used for the RNAi. Deaths occurring within 5 d after injection were considered as injection injury and excluded from the data analyses (less than 10%). Seven days after the emergence of the dsRNA-injected beetles, four adults were collected for RNA extraction to assess the RNAi efficiency by both qPCR and regular RT-PCR. qPCR for RNAi efficiency determination was conducted, as mentioned previously. RT-PCR was also carried out with 35 cycles for the target gene *TcOctβ2R* and 30 cycles for the reference gene *TcRPS3*.

### 4.7. Mobility Assay after RNAi

Locomotory responses of *T. castaneum* were measured using Syntech TrackSphere LC-300 (Syntech, Hilversum, The Netherlands). The locomotion assay was operated according to the user manual of Syntech LC-300 and a previous description [49]. Before the operation, the illumination and contrast were properly adjusted using the dark spot on a piece of paper, which could be evaluated on the video. The beetle was effectively placed in the same position of a 30 cm diameter sphere relative to the zoom lens, which served as a detector by projecting a beam of ordinary light onto the beetle. As soon as the beetle started walking, the sphere rotated in the opposite direction at the same speed as the beetle by the rotation of two motors. The beetle’s position was recorded every second, and the walking speed and direction were calculated. For the mobility assay, 7-d-old virgin adults were used. Each beetle was tested for 2 min, the average speed of the individuals was calculated. At least 40 beetles in each dsRNA-injected group were tested. The data were subjected to an independent *t*-test.

### 4.8. Mating Behavior and Fecundity Assay after RNAi

After dsRNA injection, the male and the female pupae were reared separately. The 7-d-old virgin adults were observed for the mating behavior and fecundity assay. Mating behavior was recorded by a SONY HDR-CX405. The video was started at 6 p.m. and lasted until 7:30 p.m. (90 min). The room temperature was controlled at 28 ± 1 °C. The females were first placed in 24-well plates. Then the males were quickly added and videotaping started. The start of mating was defined as when the male first mounted the back of the female in the same direction. Correspondingly, when the male left the female’s back, mating was judged to be ended. The duration of mating and the number of copulations were recorded by watching videos. The dsRNA-injected females were mated with the same dsRNA-injected males. Each dsRNA-injection group used 28 pairs of beetles to observe the mating behavior. To detect the effect of silencing of *TcOctβ2R* on fecundity, *dsGFP/dsOctβ2R-injected* females were paired with *dsGFP/dsOctβ2R*- injected males, respectively. Eggs from 3 d oviposition periods were collected/counted, and eggs were counted until the 9th day. There were 14–15 pairs of beetles in each mating group.

### 4.9. Statistical Analysis

GraphPad Prism version 8.0.1 (www.graphpad.com, access on 29 June 2021) was used for statistically analyzing and creating test graphs.

## Data Availability

Data is contain within the article or supplementary material.

## Supplementary Materials

The following are available online at https://www.mdpi.com/article/10.3390/ijms22147252/s1.
