# Supplementary material for: Knockdown of a β-Adrenergic-Like Octopamine Receptor Affects Locomotion and Reproduction of Tribolium castaneum
Source: Int J Mol Sci. 2021 Jul 6;22(14):7252. doi: 10.3390/ijms22147252 (PMC8305664; doi:10.3390/ijms22147252)
Supplement: Supplementary file 1 [file ijms-22-07252-s001.zip › ijms-1269927-supplementary.pdf]

Table S1. sequence of primers.

Application of Primers	Primer Names	Sequence (5' - 3')	Product Size(bp)
ORF cloning	TcOctβ2R-F1	CTCTT-GACTTTCTTCGACAAG	1326bp
	TcOctβ2R-R1	GCAGACTAGAAACTAG-TGGTCG	
	TcOctβ2R-F2	GCGATGGACAACTTAACATAC	1257bp
	TcOctβ2R-R2	CTGCTAGTTAGGTC-TACTCAGAGAC	
qRT-PCR	qTcOctβ2R-F	GAGCG-GAATCTCAACAAAATG	279bp
	qTcOctβ2R-R	GCGGCACAAAC-TACAAAAAAC	
qRT-PCR (reference genes)	qTcRPS3-F	CGCATTCATGGTTGATAACG	150bp
	qTcRPS3-R	GGGCACCAAGTTAGTCTGGA	
dsRNA synthesis	dsTcOctβ2R-F	taatacgactcactatagggCGTGCAC-GATCATGATCTAC	439bp
	dsTcOctβ2R-R	taatacgactcac-tatagggCTTCCCTGAAGTCCTTG	

The lowercase letters represent the T7 promoter sequences for efficient *in vitro* transcription in dsRNA synthesis.

Table S2. Overview information of the 39 sequences in phylogenetic tree.

Abbreviations	Species	GenBank Accession Number
TcOctβ1R	*Tribolium castaneum*	NP_001280514.1
TcOctβ2R	*Tribolium castaneum*	NP_001280501.1
TcOctβ3R	*Tribolium castaneum*	NP_001280505.1
TcTyrR	*Tribolium castaneum*	NP_001164312.1
DmOamb	*Drosophila melanogaste*	NP_732542.1
*DmOctα2SR**	*Drosophila melanogaste*	NP_001262714.1
*DmOctα2LR**	*Drosophila melanogaste*	NP_650754.2
DmOctα2LR	*Drosophila melanogaste*	NP_001262715.1
DmOctβ1R	*Drosophila melanogaste*	NP_001262843.1
DmOctβ2R	*Drosophila melanogaste*	NP_001303505.1
DmOctβ3R	*Drosophila melanogaste*	NP_001034046.3
DmTyrR1	*Drosophila melanogaste*	NP_524419.2
DmTyrR2	*Drosophila melanogaste*	NP_001287382.1
DmTyrR3	*Drosophila melanogaste*	NP_650651.1
BmOctα1R	*Bombyx mori*	NP_001091748.1
BmOctβ2R	*Bombyx mori*	NP_001171666.1
BmOctβ1R	*Bombyx mori*	XP_037876565.1
BmTyrR1	*Bombyx mori*	NP_001037504.1
BmTyrR2	*Bombyx mori*	NP_001164649.1
AmOctα1R	*Apis mellifera*	NP_001011565.1
AmOctβ1R	*Apis mellifera*	XP_397139.3
AmOctβ2R	*Apis mellifera*	XP_006558130.1
AmOctβ3R-1	*Apis mellifera*	XP_006557728.1
AmOctβ3R-2	*Apis mellifera*	XP_006557730.1
AmTyrR1	*Apis mellifera*	NP_001011594.1
AmTyrR2	*Apis mellifera*	NP_001032395.1
CsOctα2SR	*Chilo suppressalis*	AIC75371.1
CsOctα2LR	*Chilo suppressalis*	AIC75370.1
CsOctβ1R	*Chilo suppressalis*	AGV79326.1
CsOctβ2R	*Chilo suppressalis*	AEO89318.1

CsTyrR1	*Chilo suppressalis*	AFG26689.1
CsTyrR2	*Chilo suppressalis*	ADK91078.1
CsOctα1R	*Chilo suppressalis*	AEQ33589.1
NlOctαR	*Nilaparvata lugens*	ATY68965.1
NlOctβ2R	*Nilaparvata lugens*	ASA47149.1
NlOctβ3R	*Nilaparvata lugens*	QBC74627.
PaOctα1R	*Periplaneta americana*	AAP93817.1
PaTyrR1	*Periplaneta americana*	SNT95699.1
DmPDFR	*Drosophila melanogaste*	NP_001284827.1
